# Revitalisation endodontic treatment of traumatised immature teeth: a prospective long-term clinical study

**DOI:** 10.1007/s40368-019-00501-0

**Published:** 2019-12-06

**Authors:** H. Nazzal, S. Ainscough, J. Kang, M. S. Duggal

**Affiliations:** 1 Department of Paediatric Dentistry, School of Dentistry, University of Leeds, Clarendon Way, Leeds, LS2 9LU UK; 2 Paediatric Dentistry Section, Department of Dentistry, Hamah Dental Centre, Hamad Medical Corporation, P.O. Box: 21954, Doha, Qatar; 3 Department of Orthodontics, Royal Liverpool University Dental Hospital, Liverpool, UK; 4 Department of Oral Biology, School of Dentistry, University of Leeds, Clarendon Way, Leeds, LS2 9LU UK; 5 Discipline of Orthodontics and Paediatric Dentistry, National University Health System, Singapore, Singapore

**Keywords:** Revitalisation endodontic technique, Immature non vital teeth, Crown discolouration

## Abstract

**Purpose:**

Continuation of root development following revitalisation endodontics (RET) has been shown to be unpredictable with lower success rates in traumatised teeth. This study reports the outcomes for RET in traumatised teeth over a review period of 4 years.

**Methods:**

A prospective uncontrolled study, where RET was performed on traumatised upper immature anterior teeth with necrotic pulps in 15 children (mean age = 8.3 years), was conducted. Patients were reviewed at 3, 9, 12, 24, and 48 months, where clinical and radiographic assessments were performed. At the last review appointment, patients and parents answered questions assessing their perception and acceptance of tooth colour change over time. McNemar’s Exact test and linear mixed model assessment were used to assess changes in pulpal electrical response and radiographic evidence of continuation of root development over time, respectively.

**Results:**

There was 83.3% healing with no significant changes in EPT responses, and no significant changes in root lengths, while significant changes in root widths (*p* < 0.05) and root apex widths (*p* < 0.001) were found over time. Twenty-five percent of patients and 33% of parents felt that there were changes in tooth colour following RET over time.

**Conclusion:**

Within the limitations of this study, traumatised teeth treated using RET showed no significant root lengthening, however, acceptable periapical healing, slow thickening of root dentinal walls, and rapid development of apical closure were evident over a period of 43 months. Using Portland cement and omitting minocycline, did not eliminate crown colour change following RET.

## Introduction

Regenerative/revitalisation endodontic therapy (RET) is currently one of the accepted treatment options of non-vital immature permanent teeth by the American Association of Endodontics (American Association of Endodontics 2016), the European Society of Endodontics (Galler et al. 2016) and the European Academy of Paediatric Dentistry (Duggal et al. 2017). The predictable periodontal healing associated with this technique together with possible root lengthening and thickening of the dentinal walls make this revitalisation technique favoured over the use of the more traditional MTA apical plug technique that usually results in healing alone (Nicoloso et al. 2016; Tong et al. 2017; Torabinejad et al. 2017). RET, therefore, has been mainly recommended in the management of teeth with very short roots (less than ½ the root length), where long-term prognosis is considerably compromised (Nazzal and Duggal 2017; Kim et al. 2018).

The success of RET in terms of root lengthening and thickening of dentinal walls has been considerably lower in studies reporting the results for traumatised teeth (Nagata et al. 2014; Saoud et al. 2014; Nazzal et al. 2018a) in comparison to those reporting its use in a mixture of traumatised and non-traumatised teeth (Chueh et al. 2009; Chen et al. 2012). Recently, Lin et al. (2017) showed that with RET, root lengthening and dentinal wall thickening were significantly higher when loss of pulpal vitality was the result of a dens evaginatus rather than dental trauma.

Interestingly, the success of traumatised RET-treated immature teeth has only been reported over a short period of time in the literature [12 months (Saoud et al. 2014; Lin et al. 2017), 19 months (Nagata et al. 2014) and 2 years (Nazzal et al. 2018a)]. Such short review periods may have contributed to the impression of low success rates with RET in terms of root lengthening and thickening of dentinal walls. More long-term data is certainly needed on the success of revitalisation in traumatised teeth.

Crown discolouration is one of the most reported post-RET-treatment side effect which is mostly related to the use of bisthmuth oxide containing material and the use of minocycline in the used tri-antibiotic intracanal medicaments (Lenherr et al. 2012; Kahler and Rossi-Fedele 2016; Nazzal et al. 2018b). Although the use of white MTA in creating a hermetic cervical seal is still recommended, the use of other biocompatible material such as Portland cement (non bisthmuth oxide containing MTA, Medcem, Weinfelden, Switzerland, CE 1250) and Biodentine (Septodont, Saint-Maur-des-Fossés, France) have been recommended to reduce post-treatment discolouration (American Association of Endodontics 2016; Galler et al. 2016). In addition, other steps such as replacing/omitting minocycline from the tri-antibiotic paste or using calcium hydroxide have also been recommended to reduce post-RET discolouration (American Association of Endodontics 2016; Galler et al. 2016).

The aim of this study, therefore, was to report the success of RET in the treatment of traumatised teeth over an extended time period (average 43 months), with a secondary aim of assessing patients’ and parents’ perception and acceptance of tooth colour changes at the final review appointment.

## Methods

Ethical approval from the National Research Ethics Service (NRES) Committee, UK and consent/assent were obtained prior to the start of the study. This study was registered with clinicaltrials.gov (trial identifier: NCT03045185).

The inclusion criteria were as follows:

1. An acceptable level of cooperation allowing treatment and assessment
2. An acceptable command of the English language
3. Patients with no relevant medical history (ASA I and ASA II)
4. Children between the ages of 6 and 16 years
5. Presence of a minimum of one immature permanent anterior tooth with clinical and/or radiographic signs of pulpal pathology, requiring endodontic management following dental trauma. Children who received initial emergency pulpal extirpation with/without calcium hydroxide dressing were accepted in this study as long as no definitive endodontic treatment was attempted (calcium hydroxide apexification, MTA apical plug, RET etc.)
6. Presence of a minimum of a third of the labial tooth surface structure following tooth restoration to allow pulp sensibility testing

A two-visit RET was performed by a single operator (HN). Where indicated, local analgesia using lidocaine 2% + epinephrine 1:80,000 was delivered followed by isolation using a rubber dam. Remnants of pulp tissues, where applicable, were then extirpated using barbed broaches and the canals were minimally cleaned and shaped to prevent further weakening of the dentinal walls. Slow irrigation with 0.5% sodium hypochlorite (NaOCl) was then performed using a double vented needle, introduced to a point 2 mm short of the radiographic apical foramen. The canal was then dried using paper points. The content of Metronidazole (100 mg) and Ciprofloxacin (100 mg) capsules (TriBiodent, Royal Victoria Infirmary, Newcastle, UK), were then mixed with distilled water and introduced into the root canal system using the plastic insert of a green cannula (VasofixR Safety, B Braun, Melsungen, Germany). The contents of the third Minocycline capsule were disposed of. The root canal orifice was then covered with a cotton pellet and the access sealed using glass ionomer cement (Fuji IX, GC corporation, Tokyo, Japan).

Following resolution of signs and symptoms of infection (2 weeks in all cases), second stage RET was performed. Plain local analgesia (no vasoconstrictor) was administered, where indicated, and the tooth isolated and re-accessed as described above. The root canal system was then irrigated using copious amounts of normal saline and dried using paper points. A sterile sharp instrument (finger spreader) was then introduced into the root canal system 2 mm beyond the working length to induce intracanal bleeding. A cotton pellet was then placed into the pulp chamber around 4 mm below the CEJ to allow formation of an intracanal blood clot. The coronal pulp chamber was then thoroughly cleaned to remove any remnants of the blood and the access cavity was hermetically sealed using three layers: Pure Portland cement (Medcem, Weinfelden, Switzerland, CE 1250), glass ionomer cement (Fuji IX, GC corporation, Tokyo, Japan), and composite resin (SpectrumTPH Compules®, Dentsply, Weybridge, UK).

Post-operative periapical radiographs were taken, after which the patient was reviewed, by the same assessor, both clinically and radiographically at 3, 9, 12, 24, and 48 months. Signs of infection such as pain, abscess formation, presence of a sinus tract, tenderness to palpation and percussion were assessed at each visit. In addition, sensibility testing using cold (ethyl chloride, Axongesic®, BTC Invest, Praha, Czech Republic) and electric pulp tests (SybronEndo, Sybron dental Specialties, California, USA) were performed.

**Fig. 1:**
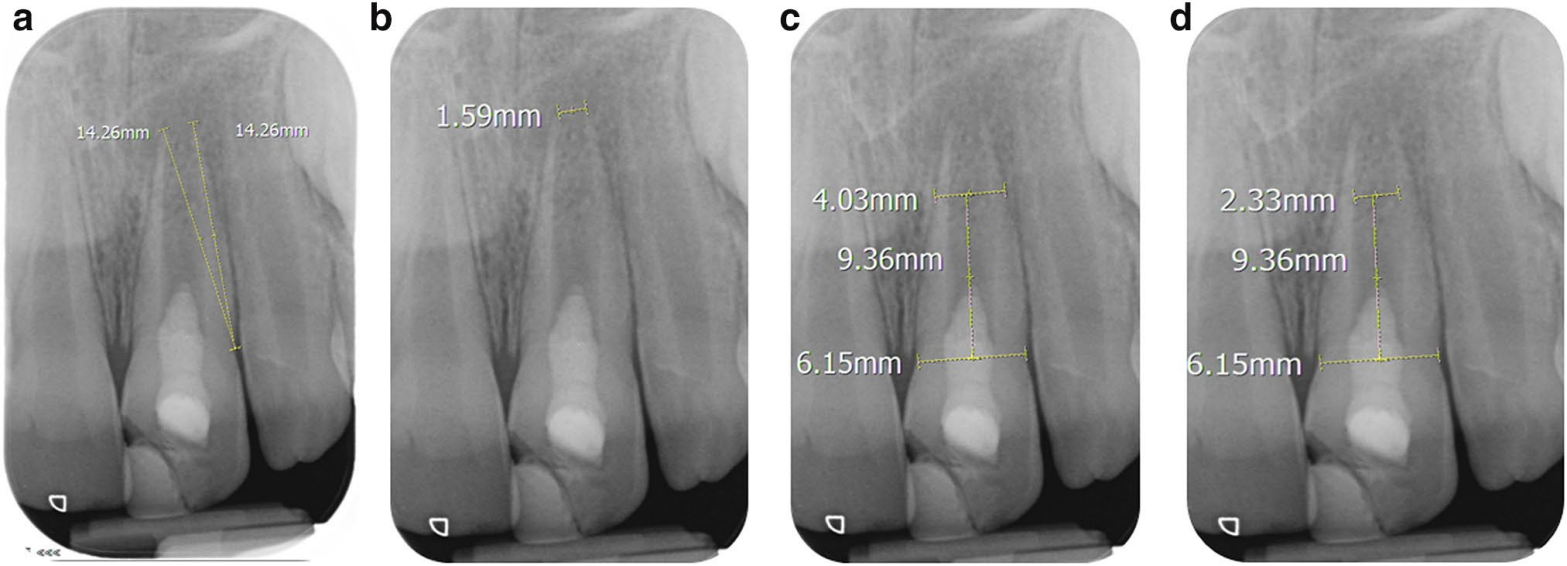
Radiographs showing root measurements (using Infinitt digital radiograph software, Seoul, Korea), **a** Root length: the distance between the cemento-enamel junction and the radiographic apex measured on mesial and distal sides of the root. The average of the mesial and distal root length measurements was recorded as the root length. **b** Apical foramen width: The distance between the mesial and distal apical ends. **c**, **d** Dentinal width: the outer root thickness and the inner pulp canal width at two-thirds root length were measured. The difference between the outer root thickness (**c**) and the inner pulp canal width (**d**) was recorded as the root dentinal wall width. Reproduced with permission from International Endodontics Journal (Nazzal et al. 2018a)

Periodontal healing was considered in cases showing a lack of clinical signs/symptoms of infection and regression/lack of progression of radiographic periapical radiolucencies. Radiographic exposures were performed by the same operator, using the same X-ray machine and technique (Jadhav et al. 2012; McTigue et al. 2013; Nagy et al. 2014; Narang et al. 2015). Radiographic standardisation was achieved using a paralleling device (Dentsply Rinn, Elgin, IL). Two calibrated investigators (HN and SA) viewed and measured the radiographs using Infinitt digital radiographic software (Seoul, Korea). A modification of the line measurement technique used by Jeeruphan et al. (2012) and Nagy et al. (2014) and described in Nazzal et al. (2018a, b) (Fig. 1) was used. Inter-examiner reliability was measured using intra-class correlation with the average of the measurements obtained by the two assessors used as the final measure for each radiographic outcome.

A growth profile was plotted over time for root lengths, root dentinal widths and apical foramen widths for the 12 individuals remaining in the study over the study period (average 43 months). A linear fit was performed using a linear mixed model, assuming individuals as random effect and changes over time as a fixed effect. Using this mixed model approach, missing data was only dropped at certain time points (as a result of missing recall appointments or imaging issues), while the remaining data of the participants was retained (Ibrahim and Molenberghs 2009). The slopes of the linear fit were reported with 95% confidence intervals and a significance level was set at 0.05. Statistical analysis was performed using the statistical package R (version 3.5.2, R Foundation, https://www.r-project.org/foundation/).

In addition, to assess the perception of colour change, patients and parents were asked a set of four questions to assess both their perception and acceptance of colour change at their final review appointment.

## Results

A summary of the participant demographics, type of trauma resulting in loss of vitality, pre-operative clinical and radiographic signs of infection, pre-operative root development stage, and reasons for exclusion are presented in Table 1. Three participants were excluded from the study due to various reasons as outlined in Table 1 leaving 12 participants with a mean age of 8.3 years (range 7–10) at the start of RET. The average recall time was 43.42 months (range 27–59 months, Table 1).

**Table 1:** Showing participant demographics, type of trauma resulting in loss of vitality, pre-operative (first RET session) root development stage, in addition to clinical and radiographic signs of infection, results of sensibility tests both pre-operatively and at last recall appointment

	Sex	Age (years)^a^	Trauma type	Root length	Presence of swelling and or pus discharge		Radiographic periapical lesion		Cold BL^b^	Cold last recall	EPT BL^b^	EPT last recall	Recall period (months)
					BL**	Last recall	BL**	Last recall					
1	M	8	E/D 11	3	No	No	Yes	No	(−)	(−)	(+)	(−)	59
2	M	7	E/D C 11	3	Sinus + pus	No	Yes	No	(−)	(−)	(−)	(−)	52
3	M	8	Avulsion 11	3	Sinus + pus	No	Unclear	No	(−)	(−)	(−)	(−)	50
4	M	10	E/D/R 11	4	Sinus + pus	No	Yes	No	(−)	(−)	(−)	(−)	27
5	M	9	E/D 22	Excluded: second trauma									
6	M	9	E/D 22	3	No	No	Yes	No	(−)	Un	(−)	(+)	46
7	M	9	E/D 11	Excluded: failed to return									
8	M	7	E/D 21	3	No	No	Yes	No	(−)	(−)	(−)	(+)	44
9	M	8	E/D 21	3	Sinus + pus	No	Yes	No	(−)	(−)	(−)	(−)	44
10	F	8	E/D 11	3	Sinus + pus	No	Yes	No	(+)	(−)	(+)	(−)	38
11	F	7	Intrusion	Excluded: apical stop									
12	M	9	E/D11	4	No	No	No	No	(−)	(−)	(−)	(−)	44
13	M	10	E/D C 11	4	No	Slight TTP	Yes	No	(−)	Un	(−)	(−)	38
14	M	9	E/D 11	3	No	Slight TTP	No	No	(−)	(−)	(+)	(+)	36
15	F	7	Avulsion 21 (2 h in milk)	2	No	No	No	No	(+)	(+)	(−)	(+)	43

^a^At start of treatment

^b^BL Baseline (At first stage RET session. Some cases received initial pulp extirpation with calcium hydroxide dressing prior to this session), *E* Enamel, *D* Dentine, root lengths: 1 = ¼ root length, 2 = ½ root length, 3 = 2/3 root length, 4 = complete root length with open apex, 5 = complete root length with closed apex, (−): Negative response, (+): Positive response, Un: unreliable response

Enamel-dentine crown fracture was the main reason for loss of vitality in the cohort of patients treated (Table 1). Signs of infection, such as the presence of apical radiolucency, and presence of a sinus tract with or without pus discharge were evident in 8 (66%), and 5 (41%) patients at baseline (first RET appointment), respectively (Table 1). Most teeth (8, 66.7%) presented with two-thirds of their root formed.

At the final recall appointment, only two cases showed evidence of slight tenderness to percussion with no other clinical or radiographic signs of infection (Table 1). No treatment was provided in these two cases; however, these patients were kept under close observation.

Only one tooth was reliably responsive to cold testing, while four teeth (33%) were responsive to electric pulp testing at the last recall appointment (Table 1). McNemar’s Exact test using binomial distribution, however, showed no significant effect with RET on eliciting an EPT pulpal response at the final review appointment (average 43 months, *p *> 0.05). An excellent overall agreement between the two assessors, as shown by a high intra class correlation score of 0.995 (95% confidence interval of 0.993 to 0.996) (Cicchetti 1994), was calculated.

**Fig. 2:**
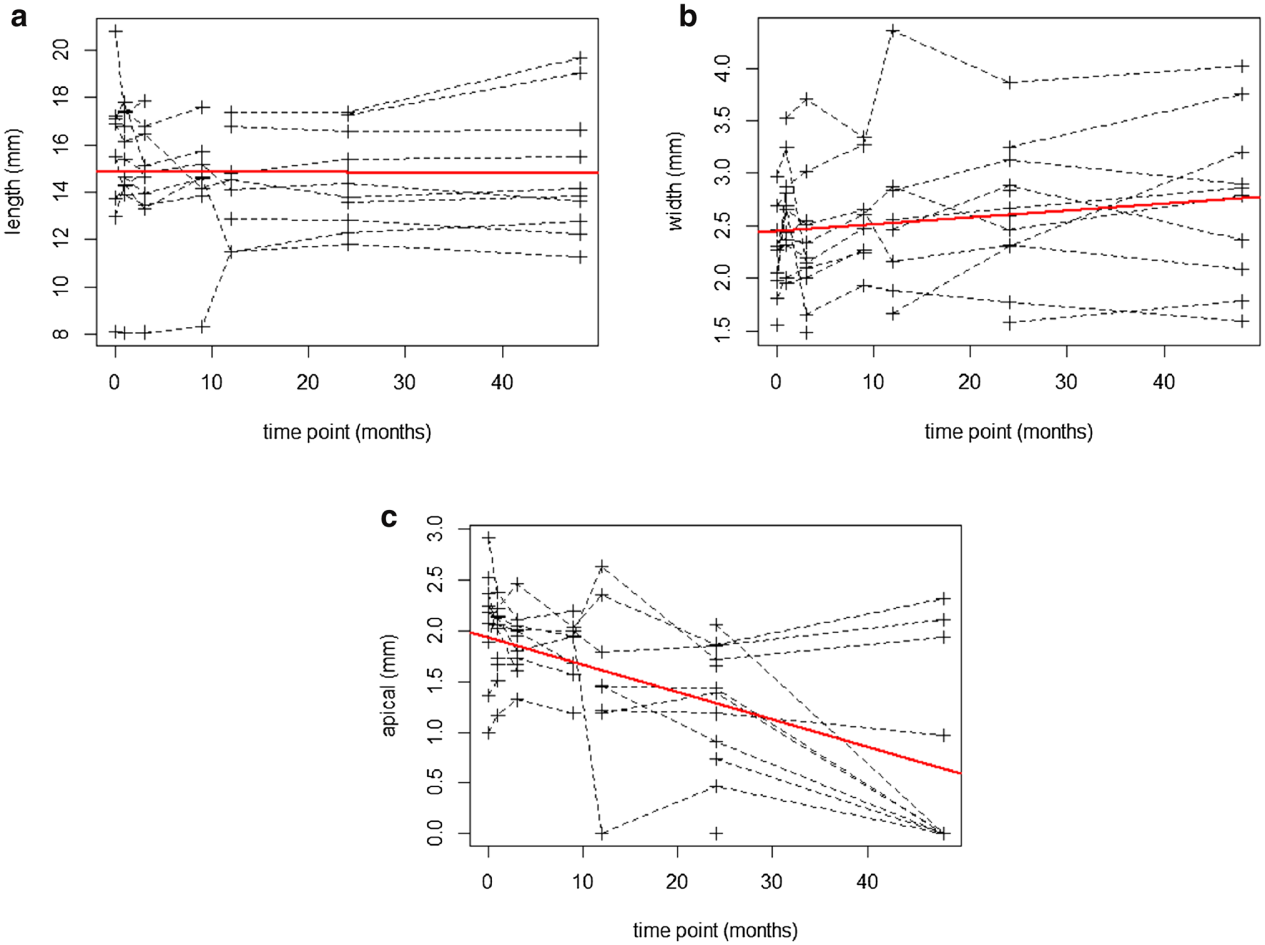
Growth profile plotted over the study period (mean 43.42 months). **a** Root length growth profile. The slope (in red) is not significant (*b *= − 0.001, 95% CI − 0.017 to 0.015), which indicate no significant change of length over time. **b** Root dentinal width growth profile. The slope (in red) is found to be significant (*b *= 0.0066, 95% CI 0.0006 to 0.0126, *t *= 2.2, *p *< 0.05), which indicate significant increase of root dentinal width over time. **c** Root apex width growth profile. The slope (in red) is significant at 0.001 significance level (*b *= − 0.027, 95% CI − 0.020 to − 0.034, *t *= − 7.6, *p *< 0.001), which indicate significant reduction of root apex width over time

The means and standard deviations of radiographic measurements at each timepoint are shown in Table 2. A root length growth profile was plotted over the study period (Fig. 2a) and a linear fit (root length = 14.88 +(− 0.001) × time in months) was performed using a linear mixed model. The slope was not significant (*b* = − 0.001, 95% CI − 0.017 to 0.015), which indicated no significant change of root length over time.

**Table 2:** Mean (± standard deviation) radiographic measurements at each timepoint in millimeters

	Baseline	Post-operative	3 months	9 months	12 months	24 months	Last recall
Root length	15.12 (3.57)	15.09 (2.73)	14.48 (2.55)	14.25 (2.66)	14.18 (2.20)	14.72 (1.93)	14.86 (2.81)
Root dentinal width	2.23 (0.44)	2.62 (0.48)	2.34 (0.62)	2.60 (0.49)	2.60 (0.83)	2.64 (0.63)	2.73 (0.80)
Root apex width	2.06 (0.59)	1.90 (0.37)	1.88 (0.30)	1.82 (0.32)	1.51 (0.80)	1.27 (0.63)	0.73 (1.01)

A root dentinal width profile was plotted over the study period (Fig. 2b) and a linear fit (root dentinal width = 2.45 + 0.0066 × time in months) was performed using a linear model. The slope was significant at 0.05 significance level (*b* = 0.0066, 95% CI 0.0006 to 0.0126, *t* = 2.2, *p* < 0.05), which indicated significant change of width over time.

A root apex width profile was plotted over the study period (Fig. 2c) and a linear fit (root apex width = 1.93 +(− 0.027) × time in months) was performed using a linear model. The slope was significant at 0.001 significance level (*b* = − 0.027, 95% CI − 0.020 to − 0.034, *t* = − 7.6, *p* < 0.001), which indicates significant change of root apex width over time.

The results of perception of tooth colour change were similar between patients and parents (Table 3). Although a third of patients and 41.6% of parents reported observing a change of colour, since initiation of RET treatment, the majority of patients, and parents were not concerned about the colour change (Table 3).

**Table 3:** Showing parent and patient’s perception of colour change of RET treated tooth at 4 year follow up

Question	Patient (*n*, %)	Parent (*n*, %)
Do you think the tooth changed colour since start of treatment?		
Yes	3 (25%)	4 (33.3%)
No	3 (25%)	3 (25%)
Not sure	4 (33.3%)	3 (25%)
Missing	2 (16.6%)	2 (16.6%)
Do you think the tooth colour is different to the contralateral tooth?		
Yes	8 (66.6%)	8 (66.6%)
No	2 (16.6%)	1 (8.3%)
Not sure/missing	2 (16.6%)	3 (25%)
What colour did it change to?		
Yellower	4 (50%)	3 (37.5%)
Darker	2 (25%)	3 (37.5%)
Greener	1 (12.5%)	0
Brown	1 (12.5%)	0
Not sure	0	2 (25%)
Would you like to get the tooth colour changed?		
Yes	4 (33.3%)	5 (41.6%)
No-patient/parent no concerned	4 (33.3%)	3 (25%)
Not applicable	2 (16.6%)	1 (8.3%)
Not sure/missing	2 (16.6%)	3 (25%)

## Discussion

Despite the unpredictability of RET outcomes, especially in terms of root lengthening and thickening of dentinal walls (Tong et al. 2017; Torabinejad et al. 2017), RET has gained popularity amongst paediatric and endodontic specialists in the past decade (American Association of Endodontics 2016; Galler et al. 2016; Duggal et al. 2017). Different hypotheses have been proposed for such unpredictability in the outcomes of this technique, such as variability in the used RET protocols (Kontakiotis et al. 2015), the lack of controlled tissue engineering scaffolds and signalling molecules used (Kim et al. 2018; Matoug-Elwerfelli et al. 2018), the effect of trauma/infection on Hertwig Epithelial Root Sheath (HERS) (Nazzal and Duggal 2017; Kim et al. 2018) and the lack of studies showing long-term results of RET treated teeth.

The success of RET has been reported in terms of healing, root lengthening, thickening of dentinal walls, apical foramen closure and tooth survival. Apart from slight tenderness to percussion in two cases, all treated teeth showed no signs of infection or progression of periapical radiolucency resulting in a periodontal healing rate of 83.3% (10/12 children). These results are lower than those reported by other researchers in the management of traumatised teeth 95.7–100% (Nagata et al. 2014; Saoud et al. 2014; Lin et al. 2017; Nazzal et al. 2018a) and represents a reduction in periodontal healing, previously reported at 2 years (Nazzal et al. 2018a). This could be a result of the extended review period presented in the current study.

The possibility of pulpal neuro-regeneration, assessed using sensibility testing, was only evaluated in 5 out of 14 studies (Tong et al. 2017) in which only 3 reported positive responses, however, at low or inconsistent levels. The 2 year results of the current study (2018) (Nazzal et al. 2018a) was the only study, where a positive response of 41.6% was reported after 2 years in RET-treated traumatised teeth. Reviewing these teeth at 43 months showed a lower response to sensibility testing. The results of sensibility tests should be interpreted with caution as, albeit very popular (Ghouth et al. 2019), are subjective, therefore, have compromised accuracy, especially in the child population. Recent systematic reviews (Ghouth et al. 2018; Mainkar and Kim 2018) showed high specificity (0.93) and low sensitivity (0.72) when using the EPT and moderate specificity (0.84) and sensitivity (0.87) for cold tests. Furthermore, the efficacy of such tests is likely to be compromised by the three-layered coronal seal which further limits their value in the assessment of pulpal neuro-regeneration.

The radiographic standardisation technique used in this study was in line with published studies (Jadhav et al. 2012; McTigue et al. 2013; Nagy et al. 2014; Narang et al. 2015), whereby a paralleling device was used by the same assessor using the same radiographic machine and setting. Other standardisation and or image calibration techniques such as the use of paralleling devices with impression bite registration (Dabbagh et al. 2012), the use of Schei’s Ruler (Santhakumar et al. 2018) and the use of TurboReg plug-in software (Bezgin et al. 2015) has been reported. The use of Cone Beam Computed Tomography has also been reported in one study (Alagl et al. 2017). To date, however, no study has compared the effectiveness of using such techniques over the standard use of paralleling techniques.

In terms of root lengthening and thickening of dentinal walls, most systematic reviews and meta-analysis have shown inconsistent results following RET ranging between 80 and 94% (depending on the scaffold used) regardless of the cause of loss of vitality (Kontakiotis et al. 2014; Tong et al. 2017; Torabinejad et al. 2017). Our study showed no significant increase in root lengths with a slow increase in root dentinal widths. Over a period of 2 years, a previous report of the patients in this study (Nazzal et al. 2018a), showed an insignificant increase in root lengths of 46.7% (7/15) and root dentinal thickness of 40% (6/12). In traumatised teeth, over a period of 12 months, Saoud et al. (2014) showed an increase in root lengths and thickening of root dentinal walls in all 20 cases observed. Applying a threshold of 20% increase in dimensions, no increase in root lengths and only 45% (9/20) increase in root dentinal thickness was reported. Over a period of 19 months, Nagata et al. (2014) reported an overall increase in root lengths in 8 teeth (34.8%) and thickening of root dentinal walls of only 10 teeth (43.5%). These results show that continuation of root development in the form of root thickening is a more likely outcome of RET in traumatised teeth than root lengthening. This finding could be attributed to the damage to HERS cells following dental trauma. Such damage is likely to affect root lengthening rather than thickening of dentinal walls which is the result of odontoblastic and cementoblastic activity.

Apical foramen closure, on the other hand, has been shown by 10 out of 14 studies with a success rate of 76–82% (depending on the scaffold used), regardless of the cause of loss of vitality (Tong et al. 2017). This finding was also evident in traumatised teeth with success rates between 60 and 100%. This outcome is the most statistically significant radiographic outcome in the current study with an average apical closure of 1.33 mm over an average period of 43 months (average 64.5% decrease in space). These results for apical closure together with no evidence of significant increase in root length indicates development of an apical barrier rather than continued apical closure.

The use of growth profile plotted over time in the current study, allowed assessment of root development over time. The results showed no significant increase in root lengths over time, while a significant increase (0.5 mm, 22.4% increase on average) in root dentinal thickness and apical closure was observed. Thickening of dentinal walls seems more achievable in traumatised teeth than root lengthening which supports the hypothesis that damage to HERS following dental trauma is a likely cause for the lack of root lengthening, while thickening of dentinal walls is a consequence of odontoblasts and/or cementum on the pulpal surfaces of root dentinal walls. Such thickening is shown to be slow with only 22% increase achieved over an average period of 43 months in the current study. Such reduction could further support the use of RET in traumatised teeth despite the lack of root lengthening as thickening of thin dentinal root walls could help strengthen such immature teeth.

With regards to tooth colour change, patients and parents were mostly unable to recall tooth colour change over time (Table 3). On the other hand, patients and parents seemed more certain when asked to compare the colour of the RET-treated tooth to that of the contralateral tooth. Comparing the tooth to the contralateral tooth, although useful in assessing tooth colour change, does not relate the change to the RET procedure as other causes could have resulted in tooth colour change such as intra coronal bleeding at the time of trauma and the presence of necrotic pulp tissues. Nevertheless, the results suggest that RET using Portland cement, instead of MTA, and biantibiotic paste, without minocycline, could also lead to noticeable tooth colour change resulting in the need for tooth colour management in four cases in comparison to only one case after 2 years (Nazzal et al. 2018a). The increased demand for tooth colour management could be a consequence of children becoming more conscious of their image with age. Currently the use of calcium hydroxide or biantibiotic paste as intracanal medicament and non-bisthmuth oxide containing bioceramic material in RET are recommended (American Association of Endodontics 2016; Galler et al. 2016) to reduce post RET crown discolouration. Clinicians, where possible, should attempt to avoid the use of materials likely to cause tooth colour change and warn patient and parents of the possibility of tooth colour change when RET is attempted (Antov et al. 2019).

One perceived limitation to the current study is the use of low sodium hypochlorite irrigant (0.5%) without immediate EDTA rinse, the lack of EDTA use at the second RET appointment, and the lack of using collagen separating the blood clot from the sealing material which are currently recommended (American Association of Endodontics 2016; Galler et al. 2016).

The use of 1.5–3% sodium hypochlorite intracanal irrigation was recommended following the work of Martin et al. (2014) comparing the effect of different sodium hypochlorite concentrations on stem cell viability. Although, the use of 0.5% sodium hypochlorite concentration was shown to have comparable effect on stem cell survival to that of the recommended 1.5–3% (Martin et al. 2014), such low concentration could reduce the antibacterial effect of sodium hypochlorite.

The use of EDTA in RET has been recommended to reduce the effect of sodium hypochlorite on stem cell viability (Martin et al. 2014) and release signalling molecules capable of promoting stem cell differentiation. EDTA is a chelating agent able to demineralize the superficial dentine layer, therefore, releasing dentinal growth factors consequently promoting stem cell differentiation into the desired odontoblasts (Galler et al. 2011).

Canal disinfection, using high concentration antibiotics, has recently been challenged due to the effect of high antibiotic concentration on stem cell survival (Diogenes et al. 2014), and the difficulties in removing antibiotics from root canal systems (Berkhoff et al. 2014). Therefore, the use of low concentration antibiotic mixtures or calcium hydroxide have been recommended by the current guidelines (American Association of Endodontics 2016; Galler et al. 2016).

The protocol used in the current study was in line with the published literature at the time of conducting this study (Kontakiotis et al. 2015), which also proved successful in achieving continuation of root development when treating earlier cases reported by our team (Abudiak et al. 2013; Nazzal et al. 2018a). Deviations from the currently recommended RET protocols were also seen in studies performed in managing immature teeth with necrotic pulps following dental trauma (Nagata et al. 2014; Saoud et al. 2014; Nazzal et al. 2018a). Such differences in RET protocols stem from the lack of high-quality research in favour of one protocol over the other, hence, the variability in the recommended RET protocols remains an issue across guidelines or even within each published guideline (American Association of Endodontics 2016; Galler et al. 2016; Duggal et al. 2017). The authors, however, recommend the use of the currently recommended protocols in future research projects.

## Conclusions

The RET protocol used in this study showed slightly lower periodontal healing and slow thickening of root dentinal walls over an average period of 43 months in comparison to the results previously published at 2 years. This study, therefore, highlights the importance of long-term assessment and reporting of RET outcomes. The slow increase in root dentinal thickness could further strengthen immature teeth. Post-RET discolouration remains an issue following RET, therefore, informed consent should be obtained prior to treatment and a clear plan for management of such colour change should be in place, prior to treatment.
